# Letter from the Editor in Chief

**DOI:** 10.19102/icrm.2019.100908

**Published:** 2019-09-15

**Authors:** Moussa Mansour


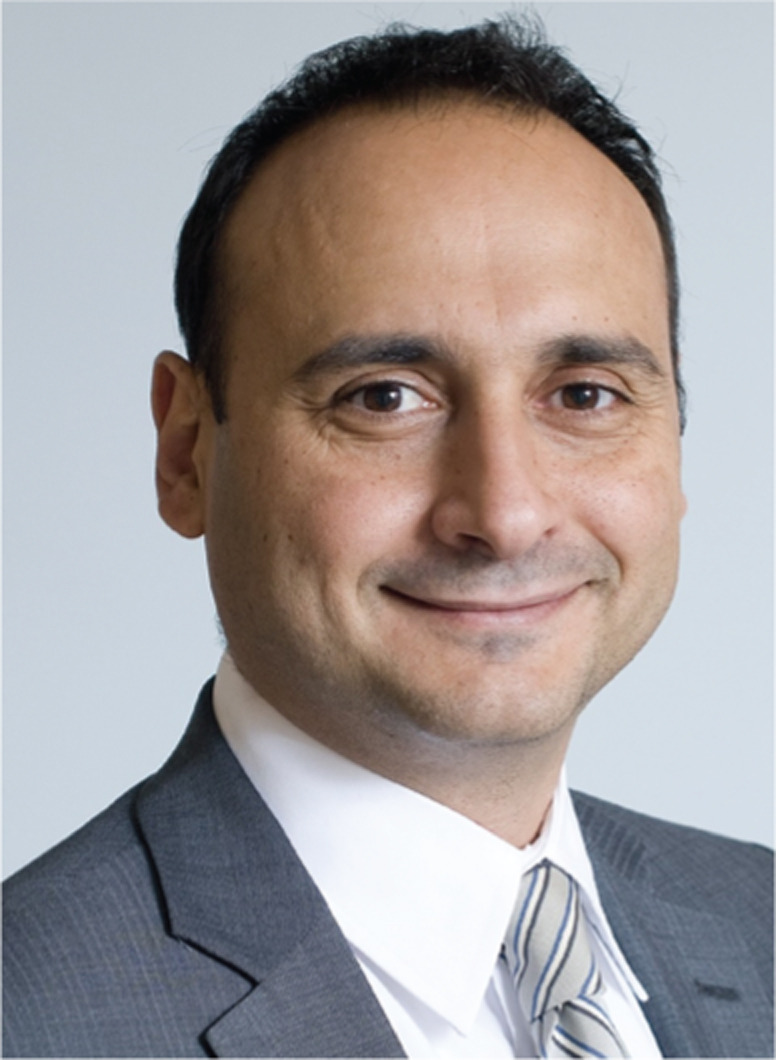


Dear Readers,

This issue of the JICRM is focused on cardiac monitoring, which has been an area characterized by rapid expansion in the past few years. One article by Amuthan et al.^1^ describes hospital inpatient monitoring as a new indication for patch monitoring. Another one, by Doshi et al.,^2^ presents a case of a consumer device used to detect atrial fibrillation (AF).

The expansion in the area of cardiac monitoring is probably related to two reasons. First, there is an increased awareness of the growing epidemic of AF in the aging United States (US) population and the understanding of the importance of reducing stroke. Second, rapid advances in technology have supported the development of monitoring tools that are smaller and lighter in addition to consumer tools that can be attached to or embedded in smartphones and watches.

Smartphones and watches are used by hundreds of millions of people in the US. They are very versatile and can be paired with devices designed for biological purposes such as the monitoring of heart rate and rhythm, sweat electrolytes, noninvasive glucose, and ultraviolet light exposure. As a result, these consumer devices may one day replace many of the medical monitoring devices currently used, such as cardiac monitors.

These consumer monitoring devices will generate huge amounts of data that will certainly have a significant beneficial impact on health care. People will more easily have access to recordings of their biological metrics such as heart rate and rhythm around the clock. This kind of information will undoubtfully result in the detection of undiagnosed conditions such as AF and likely to improve health care. One challenge, however, is the need to absorb the explosive amount of information being received from consumer devices. The current infrastructure of most medical facilities is not suited to handle the heavy amount of information submitted by patients, many of whom have direct communication lines with their providers via electronic medical record systems. As a result, automated signal analysis tools and machine learning must be developed further to automatically analyze the recorded data and filter out irrelevant information.

I hope that you enjoy reading this issue and find its content to be educational.

Sincerely,


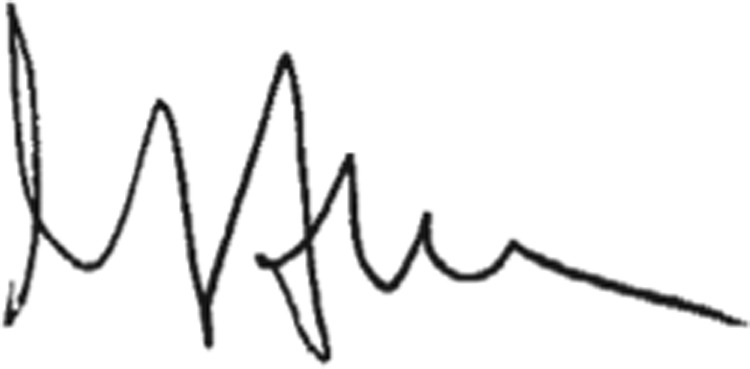


Moussa Mansour, MD, FHRS, FACC

Editor in Chief

The Journal of Innovations in Cardiac Rhythm Management

MMansour@InnovationsInCRM.com

Director, Atrial Fibrillation Program

Jeremy Ruskin and Dan Starks Endowed Chair in Cardiology

Massachusetts General Hospital

Boston, MA 02114
